# Analysis of the status quo and clinical influencing factors of the social cognitive impairment in deficit schizophrenia

**DOI:** 10.3389/fpsyt.2024.1470159

**Published:** 2024-10-02

**Authors:** Huang Chengbing, Wang Jia, Zhuang Lirong, Zhu Tingting, Song Yanling, Sun Taipeng, Zhang Xiangrong

**Affiliations:** ^1^ Department of Geriatric Psychiatry, The Affiliated Brain Hospital of Nanjing Medical University, Nanjing, China; ^2^ Department of Psychiatry, Huai’an No.3 People’s Hospital, Huaian, China; ^3^ Department of Psychiatry, Lianyungang Rehabilitation Hospital, Lianyungang, China

**Keywords:** deficit schizophrenia, eye complex emotion discrimination task, game of dice task, Iowa gambling task, social cognitive impairment, neurocognitive functioning, clinical influencing factors

## Abstract

**Background:**

Due to the high heterogeneity of schizophrenia, the factors influencing social cognitive impairment are controversial. The purpose of this study was to investigate the social cognitive dysfunction of deficit schizophrenia (DS), and to explore its clinical impact on the clinical characteristics and neurocognitive function assessment results.

**Methods:**

This study involved 100 DS patients, 100 non-deficit schizophrenia (NDS) patients, and 100 healthy controls (HC). Social cognitive functions were assessed using the Eye Complex Emotion Discrimination Task (ECEDT), Game of Dice Task (GDT), and Iowa Gambling Task (IGT), while neurocognitive functions were examined using the Clock Drawing Task (CDT), the Verbal Fluency Task (VFT), Digit Span Test (DST), Stroop Color-word Test (SCWT), and Trail Making Test (TMT). We analyzed the differences in cognitive function among the three groups of patients and the correlation between cognitive function assessment results and Positive and Negative Syndrome Scale (PANSS) scores.

**Results:**

Comparison of neurocognitive functions among the three groups through CDT, VFT, DST, SCWT, and TMT revealed that in the values of these tests in the DS group differed significantly from those of the NDS and HC groups. However, the DSB of the NDS group was lower and the TMT results were significantly higher than those of the HC group. In the DS group, ECEDT emotion recognition was positively correlated with stroop colors and stroop interference; the score of gender recognition was positively correlated with VFT, DSF, and SCWT, and TMT-B; the total time spent was positively correlated with TMT; The GDT risky option was negatively correlated with VFT, DST, stroop word, and stroop interference; the negative feedback utilization was negatively correlated with PANSS-Negative; TMT was positively correlated with VFT; IGT was positively correlated with CDT, VFT, DST, and SCWT, but it was negatively correlated with PANSS-Negative and TMT, with statistically significant.

**Conclusion:**

There are significant social cognitive impairments in the perception of social information, judgment and resolution of social problems in deficit schizophrenia, which are closely related to negative symptoms and multidimensional neurocognitive dysfunction such as attention, learning, memory, brain information processing speed, cognitive flexibility, and functional executive power.

## 1 Introduction

Social cognition is an advanced cognitive process through which individuals recognize social objects (human inner world, interpersonal relationships, social groups, social norms) in the social environment and then make judgments and speculations to adjust their adaptive behaviors, such as both social information perception and social problem judgment and resolution (1). Social cognitive impairment, compared with neurocognitive impairment, may be more closely associated with social dysfunction in patients with schizophrenia. Using social cognitive impairment as an entry point to study the pathogenesis, intervention strategies, and clinical prognosis of schizophrenia is an important avenue to explore the prevention and treatment strategies of the disease in recent years (2, 3). Unfortunately, confined to the highly heterogeneous nature of schizophrenia, there are too many confounding factors in the relevant studies, and there is a lack of consistent research findings (4).

Deficit schizophrenia (DS) is a highly homogeneous and independent disease subtype of schizophrenia spectrum disorders, with the basic clinical trait being prominent primary negative symptoms and persistence (5). Compared with non-deficit schizophrenia (NDS), DS is characterized by familial aggregation, a higher summer birth rate, a higher proportion of males, poor clinical outcomes, an early decline in social functioning. DS also has a higher rate of recognition and long-term stability in follow-up studies, making it more likely to cause reproducible and consistent conclusions (6). Therefore, in this study, we used DS patients as research subjects, observed their social cognitive decline, compared these traits with NDS patients and healthy individuals, and explored the clinical influencing factors of social cognitive impairment in patients with DS by combining clinical symptoms and the results of neurocognitive function assessment.

## 2 Objects and methods

### 2.1 Selection of study subjects

This study included 200 patients with schizophrenia who were hospitalized at Huai’an No. 3 People’s Hospital from August 2020 to December 2023. The inclusion criteria were: ① meet the diagnostic criteria of schizophrenia (F20) in the International Classification of Diseases, 10th edition (ICD-10), ② age of 18-59 years, ③ right-handed, ④ no speech or visual-auditory disorders, and the ability to cooperate in completing the tests and examinations. The exclusion criteria were: ① pregnant and lactating women,② Intellectual Disability or epilepsy, craniocerebral tumor, psychoactive substance abuse, ③ obvious side effects of drugs, ④ history of electroconvulsive therapy in the last 6 months. After enrollment, the study subjects were differentiated into DS group or NDS groups according to the Deficit Schizophrenia Diagnostic Scale (5), with 100 cases in each group. When the sample size of one group met the criteria, it was not enrolled again, while in the other group, the study subjects were continually included until the completion of the design requirements.

HC group: Healthy adults were recruited openly from the society, and the enrollment criteria were: ① no history of psychiatric or neurological diseases, ② psychoactive substance abuse or dependence, ③ no history of serious physical diseases and traumatic brain injury, ④ psychiatric diseases in their first-degree relatives, ⑤ sex, age (difference with DS group ≤2 years), and the level of education matched 1:1 with the DS group; ⑥ right-handedness, and ⑦ informed and voluntary participation in this study, and able to cooperate in completing the tests and examinations. The exclusion criteria for this group were the same as those of the schizophrenia group.

The sample size calculation was performed using PASS 2023. We used the findings of Robyn Langdon et al., 2014 to calculate the required sample size. The type 1 and 2 errors were set at 0.05 and 0.1, a P-value of <0.05 was considered to be significant. Our power analysis suggested that finding a significant difference in measurement would require a total sample size of 60 participants. To increase the statistical power, the number of patients per group was increased to 100.

The study followed the basic principles of the Declaration of Helsinki and the relevant regulations of the Ethics Committee of the Third People’s Hospital of Huai’an, and was reviewed and approved by the Ethics Committee of the hospital to be carried out (Ethics Approval No. 2020-014), with each patient and his/her guardian signing an informed consent form in writing.

### 2.2 Collection of demographic and clinical data

At the time of enrollment, demographic and clinical data, including name, gender, age, years of education, marriage, family history, age of onset, duration of disease, Positive and Negative Syndrome Scale (PANSS) scores, and history of medication were obtained from both groups, while status of marriage, family history, and other demographic data were collected from the HC group.

### 2.3 Social cognitive function assessment

Information on the eye complex emotion discrimination task (ECEDT) (7), Game of Dice Task (GDT) (8), and Iowa Gambling Task (IGT) (9) were used for the Assessment of social cognitive functioning.

The ECEDT is used to measure the ability to perceive information on emotions of others. The computer sequentially presented 34 photographs of emotions expressed in the eye area of Han Chinese people taken from movie and television materials. The task involved differentiation between gender and emotion (selecting the correct one from the four alternatives provided). The first four sheets in the experiment were used for practice, and the last 30 sheets were employed as the formal test. For each correct judgment, one point was awarded, and the test was for a total of 60 points.

In explicit risk situations, GDT is employed to evaluate decision-making functions. Subjects were provided with a starting capital of $1000 in virtual coins and needed to win the maximum amount of money possible by throwing a dice in 18 chances. Before each color cast, the subject could choose: a single number (the probability of winning 1/6 and the risk of profit/loss ±1000), two numbers (the probability of winning 2/6 and the risk of profit/loss ±500), three numbers (the probability of winning 3/6 and the risk of profit/loss ±200), and four numbers (the probability of winning 4/6 and the risk of profit/loss ±100); if the result of color casting occurred in any of the selected single numbers or combinations of numbers, the subject was considered to win. If the result of the color cast occurred in any single number or combination of numbers, the subject was awarded a win for the trial, and vice versa. Options with 1-digit and 2-digit are risky, and those with 3-digit and 4-digit are safe. The experimental analysis included the following metrics: risky option and negative feedback utilization (the number of times subjects selected the safe option based on the loss due to the risky choice as a proportion of the total number of choices; only those subjects who selected the risky option and had a minimum of one negative feedback were included in the statistics).

The IGT primarily assesses decision-making functions in situations involving ambiguous risk. Subjects were asked to obtain the maximum revenue possible by selecting cards. However, they were not informed in advance of the relevance of each card and the total number of times it was drawn. Every subject has an initial capital of 2000 yuan and 100 chances to draw the card. The subject selected one card at a time from any of the four card stacks (A, B, C, and D) presented on the screen, and then the reward and punishment results for the card were informed to the subject in a pre-programmed feedback manner by a computer. Cards A and B resulted in high rewards and high penalties, and were considered unfavorable choices. Cards C and D resulted in low rewards and low penalties, and were considered favorable choices. For the evaluation, every 20 choices were designated as a block, and the number of favorable choices minus unfavorable choices (C+D)-(A+B) was calculated for each block. The overall trend whether there was a social learning effect was also determined, i.e., a trend of gradually increasing favorable choices.

### 2.4 Neurocognitive functioning assessment

For neurocognitive function assessment, the following were applied: the Clock Drawing Task (CDT) (10), the Verbal Fluency Task (VFT) (11), the Digit Span Test (DST) (12), the Stroop color-word interference test (SCWT) (13), and the Trail Making Test (TMT) (14). Among these, the CDT uses the commonly employed 4-point scale to evaluate executive function, and VFT results are used to assess cognitive domains in executive function, attention, learning, and memory. Likewise, the DST reflects the memory capacity of the subject through observational indexes, including digit span forward (DSF) and digit span backward (DSB), etc. The SCWT evaluates the working memory capacity of the subject, and the observational indexes include the stroop word, stroop color, and stroop interference. The TMT includes two sub-tests, A and B. The TMT-A reflects the information processing speed of the subjects’ brain, and the TMT-B test reflects the cognitive flexibility of the subject in terms of number-letter linking time.

### 2.5 Statistical analyses

Statistical analysis was performed using SPSS 24.0 software, and the measured data were expressed as (mean ± standard deviation). Measurement information conforming to normal distribution was analyzed by independent samples t-test to compare two groups, while a one-way analysis of variance was used for three-group comparisons. Bonferroni analysis was used for the *post hoc* test. The chi-square test was used for comparisons between groups of count data. Pearson correlation analysis was applied to compare the correlation between social cognitive functioning, neurocognitive functioning, and PANSS scores. The difference at *p* < 0.05 was considered statistically significant (bilateral).

## 3 Results

### 3.1 Comparison of demographic and clinical data between deficit schizophrenia and non-deficit schizophrenia groups

Compared with the NDS group, the DS group had more number of male patients and positive family history, earlier age of onset, longer disease duration, lower PANSS positive symptom scores, and higher negative symptom score value; the differences were statistically significant at *p* < 0.05, whereas there were no statistically significant differences between age, marriage, years of education, the PANSS general pathology score, and the total score (P > 0.05). For details, see **Table 1**.

**Table 1:** Demographic and clinical information between deficit schizophrenia (DS) and non-deficit schizophrenia (NDS) groups.

		DS (n=100)	NDS (n=100)	X^2/^t	*p*
Sex	male	69	54	4.751	0.029
	Female	34	46		
Age (years)		38.93 ± 9.66	38.48 ± 9.68	0.329	0.742
Marriage	married	33	34	0.022	0.881
	unmarried	67	66		
Education (years)		10.45 ± 2.55	10.78 ± 2.72	0.885	0.377
Family history	yes	16	6	5.107	0.024
	no	84	94		
Age of onset (years)		32.96 ± 9.71	35.61 ± 9.78	1.923	0.046
Duration of disease (months)		73.56 ± 16.06	35.74 ± 11.68	19.05	<0.001
PANSS	positive	12.68 ± 5.11	22.58 ± 5.27	13.49	<0.001
	negative	29.51 ± 6.25	21.88 ± 7.08	8.078	<0.001
	general pathology	42.54 ± 7.48	41.52 ± 8.15	0.922	0.358
	total	84.73 ± 14.13	85.98 ± 15.22	0.602	0.548

### 3.2 Comparison of the three groups in terms of the results of social cognitive function assessment

Compared with the HC group, patients in the DS and NDS groups had significantly declined observational indexes, including the score of ECEDT emotion recognition and gender recognition, the GDT negative feedback utilization, and the scores of each Block of the IGT (**Table 2**). Concurrently, there was a significant increase in the GDT risky options. Likewise, compared to the NDS, the DS showed a significant decline in ECEDT emotion recognition score, the utilization rate of GDT negative feedback and the Block2-4 of the IGT. The DS risky options was also higher than NDS, with a statistically significant difference (*p* < 0.05).

**Table 2:** Comparison of social cognitive functioning ratings between the three groups.

	DS	NDS	HC	p (DS-NDS-HC)	p (DS-NDS)	p (DS-HC)	p (NDS-HC)
ECEDT							
emotion recognition	24.71 ± 2.55	26.72 ± 3.72	27.98 ± 3.07	<0.001	<0.001	<0.001	0.015
gender recognition	29.84 ± 1.28	29.85 ± 1.90	30.69 ± 1.54	<0.001	1.000	0.001	0.001
total time spent	174.7 ± 28.85	157.4 ± 26.90	138.1 ± 28.85	0.135			
GDT							
risky options	8.68 ± 2.48	7.43 ± 2.14	5.31 ± 1.68	<0.001	<0.001	<0.001	<0.001
negative feedback	57.20 ± 22.31	66.50 ± 23.84	83.18 ± 19.94	<0.001	0.009	<0.001	<0.001
IGT							
Block 1	6.39 ± 2.28	6.82 ± 1.62	8.49 ± 1.51	<0.001	0.297	<0.001	<0.001
Block 2	3.96 ± 1.63*	8.32 ± 1.95*	9.38 ± 1.77*	<0.001	<0.001	<0.001	0.012
Block 3	7.26 ± 2.39*	9.09 ± 1.65*	10.51 ± 1.19**	<0.001	<0.001	<0.001	<0.001
Block 4	4.30 ± 1.40**	10.06 ± 1.81**	11.10 ± 0.99**	<0.001	<0.001	<0.001	0.029
Block 5	8.14 ± 2.31**	10.36 ± 1.99**	11.45 ± 1.32**	<0.001	<0.001	<0.001	<0.001

Compared to Block1, *p < 0.05; **p < 0.01.

Further analysis of the IGT decision-making process of patients in the three groups revealed a progressive effect between the different Block scores in the HC and NDS groups, and the scores of Block 2 to Block 5 were significantly higher than that of Block 1 (*p* < 0.05). In contrast, although there were also statistically significant differences between the different Block scores in the DS group (*p* < 0.05), the effect was not progressive.

### 3.3 Neurocognitive function assessment of the three groups and their comparison

A comparison revealed a significant difference in the observed indexes of CDT, VFT, DST, SCWT, and TMT of patients in the DS group from those of the NDS group and the HC group, while the NDS group had a lower DST inverse order score and a higher TMT score than that of the HC group, with a statistically significant difference (*p* < 0.05) (**Table 3**).

**Table 3:** Comparison of neurocognitive functional rating among three groups.

	DS	NDS	HC	*P* (DS-NDS-HC)	*P* (DS-NDS)	*P* (DS-HC)	*P* (NDS-HC)
CDT	2.97 ± 1.12	3.73 ± 0.53	3.72 ± 0.57	<0.001	<0.001	<0.001	1.000
VFT	19.63 ± 8.64	24.04 ± 5.77	24.54 ± 4.95	<0.001	<0.001	<0.001	1.000
DST							
DSF	6.59 ± 3.81	9.58 ± 2.22	10.41 ± 1.88	<0.001	<0.001	<0.001	0.104
DSB	5.33 ± 1.49	8.48 ± 1.94	9.60 ± 1.86	<0.001	<0.001	<0.001	0.006
SCWT							
stroop word	26.75 ± 11.27	32.30 ± 10.28	33.74 ± 8.68	<0.001	<0.001	<0.001	0.947
stroop color	23.09 ± 10.15	33.22 ± 9.62	34.54 ± 9.13	<0.001	<0.001	<0.001	1.000
stroop interference	18.99 ± 9.38	30.19 ± 12.66	30.67 ± 10.69	<0.001	<0.001	<0.001	1.000
TMT							
TMT-A	24.42 ± 6.11	19.16 ± 4.49	14.88 ± 3.11	<0.001	0.001	<0.001	0.007
TMT-B	44.01 ± 5.83	33.21 ± 9.50	23.30 ± 4.94	<0.001	0.001	<0.001	0.004

### 3.4 Correlation analysis between social cognitive and neurocognitive functions and PANSS scores of the deficit schizophrenia group

Person correlation analysis showed a mild-positive correlation between the score of ECEDT emotion recognition in the DS group with stroop colors (r=0.239, p=0.017) and stroop interference (r=0.210, p=0.036). The score of gender recognition by the DS group showed a moderately-positive correlation with VFT (r=0.257, p=0.001), DSF (r=0.427, p<0.001; 0.407, p<0.001), and SCWT (r=0.351, 0.428, 0.286, p<0.05), and TMT-B (r=-0.202, p=0.044). The total time spent showed a moderately-positive correlation with TMT (r=0.367, p<0.001; r=0.238, p=0.017). The GDT risky option of the DS group exhibited a moderately-negative correlation with VFT (r=-0.431, p<0.001), DST (r=-0.225, p=0.024; r=-0.280, p=0.005), stroop word (r=-0.212, p=0.034), and stroop interference (r=-0.281, p=0.005). The negative feedback utilization by the DS group was mild-negatively correlated with PANSS-Negative (r=-0.233, p=0.002), TMT (r=0.208, p=0.037; r=0.207, p=0.038) and mild-positively correlated with VFT (r=0.199, p=0.048). IGT showed a mild-to-strongly positive correlation with CDT, VFT, DST, and SCWT (r=0.229 ~0.741, p<0.05) and moderately-negative correlation with PANSS-Negative and TMT (r=-0.206 ~-0.341, p<0.05) (**Table 4**).

**Table 4:** Correlation analysis between social cognitive and neurocognitive functioning and PANSS scores in schizophrenia.

	ECEDT			GDT		IGT				
	emotion recognition	gender recognition	total time spent	risky options	negative feedback	Block1	Block2	Block3	Block4	Block5
PANSS-Positive	-0.094	-0.034	-0.004	0.112	-0.037	-0.007	0.138	0.049	0.17	0.074
PANSS-Negative	0.072	-0.043	0.077	0.071	-0.233^*^	-0.273^**^	-0.114	-0.206^*^	-0.096	-0.227^*^
PANSS-General	-0.028	0.092	0.117	-0.005	-0.134	-0.05	-0.012	-0.066	0.032	-0.072
PANSS-total	-0.017	0.017	0.095	0.069	-0.187	-0.15	-0.007	-0.108	0.036	-0.112
CDT	0.117	0.13	-0.062	-0.036	-0.126	0.300^**^	0.091	0.229^*^	0.077	0.266^**^
VFT	0.101	0.257^**^	0.001	-0.431^**^	0.199^*^	0.493^**^	0.538^**^	0.622^**^	0.435^**^	0.574^**^
DSF	0.177	0.427^**^	0.053	-0.225^*^	0.032	0.610^**^	0.523^**^	0.708^**^	0.555^**^	0.685^**^
DSB	0.134	0.407^**^	0.073	-0.280^**^	0.135	0.593^**^	0.495^**^	0.741^**^	0.480^**^	0.657^**^
stroop word	-0.026	0.351^**^	-0.192	-0.212^*^	0.094	0.425^**^	0.388^**^	0.431^**^	0.341^**^	0.425^**^
stroop color	0.239^*^	0.438^**^	-0.075	-0.171	0.029	0.506^**^	0.470^**^	0.564^**^	0.425^**^	0.614^**^
stroop interference	0.210^*^	0.286^**^	-0.074	-0.281^**^	-0.04	0.411^**^	0.449^**^	0.469^*^	0.406^**^	0.550^**^
TMT-A	-0.075	-0.148	0.367^**^	-0.012	-0.208^*^	-0.051	-0.296^**^	-0.187	-0.311^**^	-0.285^**^
TMT-B	-0.07	-0.202^*^	0.238^*^	-0.004	-0.207^*^	-0.053	-0.341^**^	-0.114	-0.340^**^	-0.202^*^

*p<0.05; **p<0.01.

## 4 Discussion

Social cognitive impairment is an independent component of cognitive dysfunction in schizophrenia, which limits the recovery of daily social functioning of the patient independently and closely from neurocognitive impairment. Social cognitive impairment may also mediate between certain neurocognitive impairments and social dysfunction, the hotspots of schizophrenia research in recent years (15, 16). In this study, to evaluate social cognitive impairment in schizophrenic patients, we chose highly homogeneous DS patients as research subjects and applied ECEDT, GDT, and IGT. Patients in both DS and NDS groups showed significant deficits in various dimensions of social cognitive functioning, with especially severe social cognitive impairments in DS patients. We applied classical neurocognitive functioning assessment paradigms, such as CDT, VFT, DST, SCWT, and TMT, to explore the social cognitive impairments in DS patients with neurocognitive functioning and their correlation with clinical symptoms, and found different degrees of correlation between negative symptoms, neurocognitive function, and social cognitive impairment. These findings suggest the potential influence of social cognitive impairment on negative symptoms and neurocognitive function.

The perception of social information mainly includes the recognition of visual social information (such as faces, gestures, body postures, movements, situations, etc.) and auditory social information (such as verbal and nonverbal sounds, pitch, speed of sound, etc.); among these, faces are the key source for social information, because the age, gender, direction of gaze, identity, and mood of the individual can be determined, so ECEDT is often used in clinical and scientific research to asses perceived dimensions of social information (17, 18). Compared with the HC group, patients in the DS and NDS groups had significantly impaired abilities of social information perception, such as emotion recognition, gender recognition, etc., with the impairment being more serious in the DS group. Meanwhile, the correlation analysis between ECEDT and NASS scores, VFT, DST, SCWT, and TMT in DS patients suggested highly negative symptoms in patients with significant neurocognitive impairments such as executive function, attention, learning, memory, and brain function. Patients with significant neurocognitive impairments, including the speed of information processing speed and cognitive flexibility, also had more severe impairments in social information perception. These results confirmed our hypothesis, which states that emotional cognitive deficits in schizophrenia are not a unique emotional impairment, but rather a general, qualitative impairment of social information perception affected by negative symptoms and neurocognitive functioning (19, 20). Social cognitive impairments cannot be readily attributed to differences in working memory, executive function, intelligence, or antipsychotic medications (21). But patients with schizophrenia exhibiting severe multidimensional negative symptoms in the emotional, social, and behavioral attributes, and those who experience a slowdown of brain’s information processing and the decrease in cognitive flexibility, are more likely to experience communication deficits in information processing and consequently have difficulty in accurately processing social information given by others. Patients’ negative experiences of social interactions combined with neurocognitive impairments in dimensions, including executive function, attention, learning, and memory, are bound to increase each day, resulting in increasingly low self-efficacy, more severe social skill problems, and worsened social dysfunction (22–25).

In our social life, we need to face risk in two different contexts, including explicit and ambiguous risk. Hence, social cognitive functioning studies on social problem judgment and solution dimensions often need to combine two tools, GDT and IGT. From the results of this study, unlike HC, individuals with schizophrenia, especially true for patients with DS, select the risk option more often in both contexts. Functional structural imaging studies of the of the brain of patients with schizophrenia have shown lesions in the frontal lobe, hippocampus, and basal nuclei, which are severe in patients with DS (26). Damage to the prefrontal lobe impairs the cognitive processing of reward/punishment, making patients have an inclination to make detrimental choices for themselves (27).

Even in explicit risk situations, people with DS rarely change their choices in the GDT, used to assess decision-making ability, even when they receive explicit negative feedback. Impairment of this decision-making ability was related to clinical symptoms, as shown by lower negative feedback utilization and more negative symptoms. The impairment was closely related to the impairment of various dimensions of neurocognitive functioning, exhibiting moderate associations between GDT risk option scores and negative feedback utilization and neurocognitive assessment ratings, such as VFT, DST, SCWT, and TMT. Decision-making itself is a complex monitoring process of self-goal-oriented strategies, attentional selection of relevant stimuli, and inhibition of irrelevant stimuli. The decision-making process involves attention, learning, memory, executive functioning, the speed of information processing, and cognitive flexibility; thus, impaired neurocognitive functioning in patients with schizophrenia directly affects decision-making ability (28).

The IGT is employed to assess decision-making ability in fuzzy risk situations. The early blocks is dominated by fuzzy decisions, whereas in the later blocks, the task switches to risky decisions because the rules may have been figured out (29). In this study, we noted a progressive effect between the Blocks of IGT scores in the NDS and HC groups, while no such performance was observed in the patients of the DS group, suggesting that the HC group has good social learning capability and can learn the laws of the options and eventually tend to assess the advantages and disadvantages of these options. The results also show that the DS patients have impaired social learning ability and are not able to learn the laws in the IGT; in addition, they are also not aware of the advantages and disadvantages of the options, although the impaired social learning ability of the patients in the NDS group was not significant. Correlation analysis showed a weakly-moderate negative correlation between IGT scores, negative symptom scores, and TMT scores, and a moderately strong positive correlation with neurocognitive function scores, including CDT, VFT, DST, and SCWT. This is partly because the content of the IGT test has an overlap with working memory, therefore, the differences in the neurocognitive functions of the subjects may impact the results of the IGT (30). On the other hand, the level of neurocognitive functions, such as the speed of information processing, cognitive flexibility, executive function, attention, learning, memory, etc., affects the ability of an individual to learn in a social context (31).

Herein, we also compared the demographic and clinical profiles of patients in the DS and NDS groups and discovered that more patients with DS were male, and had a positive family history, an earlier age of onset and longer duration of disease, and milder positive symptoms and more negative symptoms. Neurocognitive assessment among the three groups revealed extensive neurocognitive impairment in patients with DS. Besides, the multidimensional measures of DS patients were significantly worse than those of patients with NDS and healthy adults, In contrast, the neurocognitive impairment of patients in the NDS group was not balanced; it was more obvious in the dimensions of memory, speed of information processing, and cognitive flexibility. We also observed that the parameters of attention and executive function of NDS patients were relatively less impaired, and there were no significant differences between them and healthy normals. This finding is consistent with the clinical features of DS, which is largely in line with our expectations and with previous studies by Viviano et al. (32–34).

## 5 Conclusion

In summary, the social cognitive impairment in the context of social information perception, as well as social problem judgment and resolution, may vary in patients with schizophrenia. Furthermore, social cognitive impairment may be more significant in patients with DS than those with NDS and are affected by negative symptoms and multidimensional neurocognitive impairments, includes attention, learning, memory, information processing speed, cognitive flexibility, and executive functions.

## 6 Shortcomings and prospects

Because of single research method, there some limitations study. Future research on the social cognitive impairment of schizophrenia patients in different periods by combining brain imaging, electrophysiology, and genetics, concurrently maintaining a strict homogeneous sample selection, will help us to understand the pathological mechanisms of schizophrenia deeply and search for better intervention strategies.

## Data Availability

The raw data supporting the conclusions of this article will be made available by the authors, without undue reservation.
